# Pulmonary Artery Intimal Sarcoma Mimicking Pulmonary Embolism: A Case Report

**DOI:** 10.5334/jbsr.4114

**Published:** 2025-11-04

**Authors:** Thomas Van Den Berghe, Robbert Mahieu, Koenraad Verstraete

**Affiliations:** 1 Ghent University Hospital, 9000 Gent, Belgium

**Keywords:** pulmonary artery intimal sarcoma, pulmonary embolism, computed tomography angiography, PET-CT, oncology

## Abstract

Pulmonary artery intimal sarcoma is a very rare and often misdiagnosed cause of pulmonary artery occlusion and progressive dyspnoea. A case of a 66-year-old man is presented, initially treated for presumed pulmonary embolism, in whom persistent intraluminal filling defects and inadequate therapeutic response ultimately led to the diagnosis of intimal sarcoma.

*Teaching point:* Pulmonary artery intimal sarcoma should be considered in cases of persistent suspected pulmonary embolism unresponsive to adequate anticoagulation or thrombolysis, with imaging features such as SUVmax, metabolic tumour volume, total lesion glycolysis, and the wall eclipsing sign helping in the differentiation between the two entities.

## Introduction

Pulmonary artery intimal sarcoma (PAIS) is a malignant tumour arising from the pulmonary artery’s intimal layer (300 cases reported). Clinical presentation frequently mimics pulmonary embolism (PE, incidence = 60–70/100,000), as both entities cause acute or progressive dyspnoea, chest pain, and right heart failure. Computed tomography pulmonary angiography (CTPA) often shows intraluminal filling defects, leading to initial misdiagnosis as thromboembolic disease. Recognition and multimodality imaging are essential, as prognosis and management differ fundamentally.

## Case Report

A 66-year-old man (1.75 m, BMI = 44 kg/m²) with cardiovascular comorbidities presented with resting and exertional dyspnoea over several months, intermittent chest discomfort, fatigue, cold extremities, and a 20 kg unintentional weight loss over a 2- to 3-year period.

**Figure 1:**
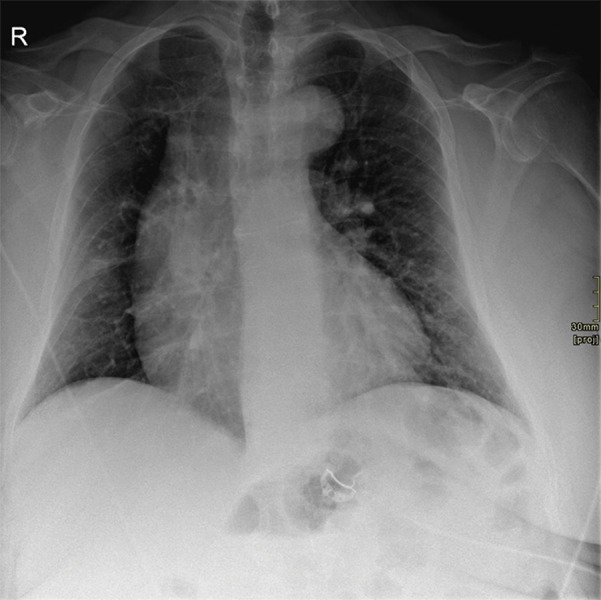
Chest radiography (frontal view) demonstrating cardiomegaly (cardiothoracic ratio = 0.59). Pulmonary vascular markings are globally increased, without evidence of consolidation.

**Figure 2:**
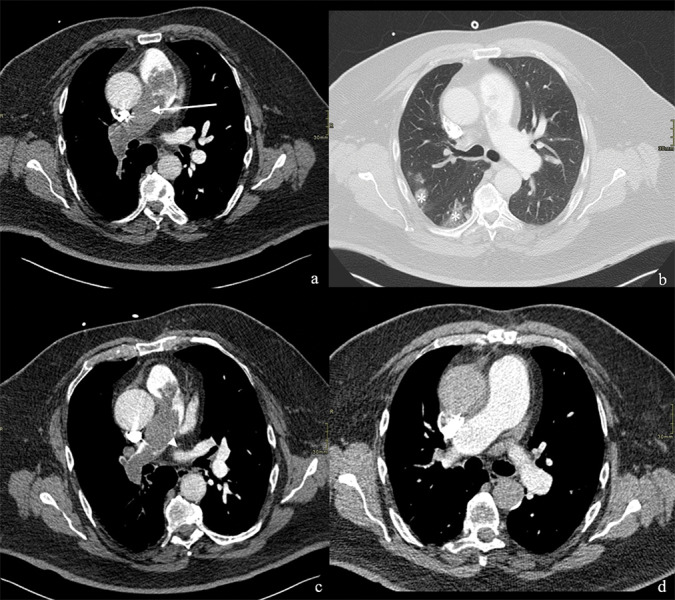
Axial CT pulmonary angiography. **(a)** Extensive intraluminal filling defects *(white arrow)* within the pulmonary trunk and right pulmonary artery. **(b)** Associated Hampton humps *(white asterisks)* in the right upper and lower pulmonary lobes, indicating pulmonary infarction. **(c)** One-week post-thrombolysis imaging demonstrating additional left pulmonary artery involvement *(white arrowhead)*. **(d)** Three months post-endarterectomy imaging showing no evidence of residual tumour.

Chest radiography showed cardiomegaly and increased pulmonary vascular markings (Figure 1). CTPA revealed extensive filling defects in the pulmonary trunk and right pulmonary artery, interpreted as massive PE with pulmonary infarctions (Figure 2a, b). Oxygen saturation was 88%. Duplex ultrasound did not demonstrate deep venous thrombosis (DVT). Anticoagulation and thrombolysis (alteplase followed by heparin) were started. Despite adequate treatment, clinical improvement remained limited.

**Figure 3:**
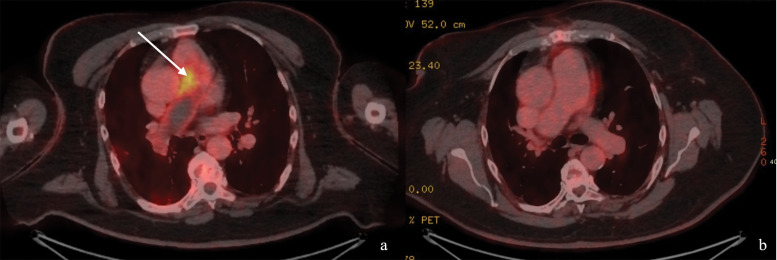
Axial ^18^F-FDG-PET-CT. **(a)** Three weeks after initial presentation, imaging demonstrating moderate metabolic activity *(white arrow)* in the wall of the pulmonary trunk (GE Medical Systems Discovery MI, PET-WB-QClear600 reconstruction, SUVmax = 6.3). **(b)** Six-month post-endarterectomy imaging showing no evidence of recurrence.

**Figure 4:**
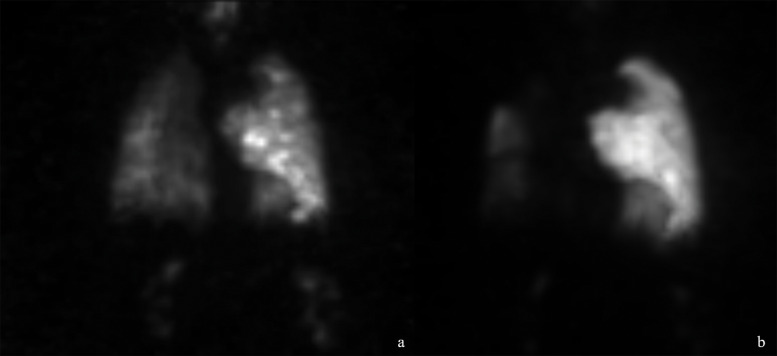
Coronal SPECT-scan taken three weeks after the initial presentation. **(a)** Ventilation imaging showing globally reduced tracer uptake in the right lung without distinct ventilation defects. **(b)** Perfusion imaging showing near-complete absence of right lung perfusion with minimal anterior preservation and mildly heterogeneous left lung perfusion without distinct defects.

The follow-up CTPA one week later demonstrated persistent expansile filling defects and new involvement of the left pulmonary artery (Figure 2c). ^18^F-FDG-PET-CT performed three weeks after presentation showed heterogeneous uptake in the pulmonary trunk wall, suspicious of malignancy (GE Medical Systems Discovery MI, PET-WB-QClear600 reconstruction, SUVmax = 6.3, Figure 3a). Ventilation–perfusion SPECT confirmed subtotal occlusion of the right pulmonary artery (Figure 4a, b). Transthoracic echocardiography revealed a mobile mass close to the pulmonary valve.

Endovascular biopsy was inconclusive. Given the progressive symptoms and imaging findings, surgical resection was performed. Intraoperatively, a friable multinodular lesion almost completely occluding the right pulmonary artery and extending into the left was noted. Subtotal pulmonary endarterectomy was performed.

Histopathology revealed atypical spindle-cell proliferation with nuclear atypia and mitotic activity with MDM2 immunopositivity, consistent with PAIS [1]. Postoperatively, dyspnoea markedly improved, oxygenation saturation stabilized at 95%, and follow-up CTPA at three months showed restored right pulmonary artery flow without residual tumour (Figure 2d). ^18^F-FDG-PET-CT at six and twelve months showed no recurrence or metastasis (Figure 3b). The patient remained clinically stable under oncological follow-up, without additional therapy.

## Discussion

PAIS is a rare tumour typically arising from the pulmonary trunk or main pulmonary arteries [2]. It often demonstrates rapid local growth, high-grade histology, MDM2 amplification, a high recurrence rate, and metastasis to the lungs, pleura, or skeletal muscle [1, 3]. Mortality is most often due to metastatic progression or vascular obstruction.

Because of its intraluminal growth, PAIS is frequently mistaken for PE. Clinical red flags include progressive symptoms despite anticoagulation, absence of DVT, unilateral involvement, and constitutional symptoms such as weight loss.

Imaging is pivotal for differentiation. On CTPA, PAIS typically appears as an expansile intraluminal mass with vessel enlargement, lobulated contours, and heterogeneous attenuation, features less typical for thrombus [4]. ^18^F-FDG-PET-CT is particularly useful: malignant lesions demonstrate increased metabolic activity, with SUVmax values significantly higher than those in thromboembolic disease [5]. Distinguishing imaging characteristics between PAIS and PE are summarized in Table 1. In our case, persistent filling defects and ^18^F-FDG uptake were key features in suggesting neoplasm.

**Table 1:** Imaging parameters and distinguishing features of PAIS versus PE across different imaging modalities.

PARAMETER	IMAGING MODALITY	PULMONARY ARTERY INTIMAL SARCOMA	PULMONARY EMBOLISM	REFERENCE
SUVmax (Mean ± SD)	PET-CT	11.1 ± 4.9 12.8 ± 14.7	1.9 ± 0.6 1.7 ± 0.3	[5, 6]
MTV (Median [IQR])	PET-CT	16.8 (11.9–29.1)	6.9 (4.1–11.0)	[7]
TLG (Median [IQR])	PET-CT	111.7 (56.5–163.3)	10.7 (5.2–16.2)	[7]
Wall eclipsing sign	CTPA	Present: lesion eclipsing the pulmonary artery wall before infiltrating outside	Absent	[8]

(CTPA = computed tomography pulmonary angiography; IQR = interquartile range; MTV = metabolic tumour volume; PET-CT = positron emission tomography–computed tomography; SD = standard deviation; SUVmax = maximum standardized uptake value; TLG = total lesion glycolysis).

Histopathology remains the gold standard for diagnosis, with MDM2 amplification serving as a supportive marker [1]. Prognosis is generally poor, with a mean survival of 17 months [9]. Reported survival is 38% and 13% at two and five years, although complete resection can extend median survival to three years [9, 10]. Adjuvant anthracycline-based chemotherapy or radiotherapy may improve disease control, but standardized protocols are lacking. Our patient showed no recurrence one year after surgery.

## Conclusion

PAIS is a rare but important differential diagnosis of PE. Persistent or atypical endovascular filling defects with a wall eclipsing sign on CTPA, together with increased SUVmax, MTV, or TLG on ^18^F-FDG-PET-CT, should raise suspicion. Early recognition and surgical resection are essential for improved outcomes.

## Figures and Tables

**Table:** 

^18^F-FDG-PET-CT	18-Fluorodeoxyglucose Positron Emission Tomography – Computed Tomography
BMI	Body Mass Index
CTPA	Computed Tomography Pulmonary Angiography
CTR	Cardiothoracic Ratio
DVT	Deep Venous Thrombosis
IQR	Interquartile Range
MDM2	Murine Double Minute 2
MTV	Metabolic Tumour Volume
PAIS	Pulmonary Artery Intimal Sarcoma
PE	Pulmonary Embolism
SD	Standard Deviation
SPECT	Single Photon Emission Computed Tomography
SUVmax	Maximum Standardized Uptake Value
TLG	Total Lesion Glycolysis
